# Effects of exercise-based cardiac rehabilitation delivery modes on exercise capacity and health-related quality of life in heart failure: a systematic review and network meta-analysis

**DOI:** 10.1136/openhrt-2021-001949

**Published:** 2022-06-09

**Authors:** Teketo Kassaw Tegegne, Jonathan C Rawstorn, Rebecca Amy Nourse, Kelemu Tilahun Kibret, Kedir Yimam Ahmed, Ralph Maddison

**Affiliations:** 1 Institute for Physical Activity and Nutrition, Deakin University, Geelong, Victoria, Australia; 2 Department of Public Health, Debre Markos University, Debre Markos, Amhara, Ethiopia; 3 Global Obesity Centre, Deakin University, Geelong, Victoria, Australia; 4 Translational Health Research Institute, Western Sydney University, Penrith, New South Wales, Australia

**Keywords:** cardiac rehabilitation, epidemiology, outcome assessment, health care, heart failure, meta-analysis

## Abstract

**Background:**

This review aimed to compare the relative effectiveness of different exercise-based cardiac rehabilitation (ExCR) delivery modes (centre-based, home-based, hybrid and technology-enabled ExCR) on key heart failure (HF) outcomes: exercise capacity, health-related quality of life (HRQoL), HF-related hospitalisation and HF-related mortality.

**Methods and results:**

Randomised controlled trials (RCTs) published through 20 June 2021 were identified from six databases, and reference lists of included studies. Risk of bias and certainty of evidence were evaluated using the Cochrane tool and Grading of Recommendations Assessment, Development and Evaluation, respectively. Bayesian network meta-analysis was performed using R. Continuous and binary outcomes are reported as mean differences (MD) and ORs, respectively, with 95% credible intervals (95% CrI). One-hundred and thirty-nine RCTs (n=18 670) were included in the analysis. Network meta-analysis demonstrated improvements in VO_2_peak following centre-based (MD (95% CrI)=3.10 (2.56 to 3.65) mL/kg/min), home-based (MD=2.69 (1.67 to 3.70) mL/kg/min) and technology-enabled ExCR (MD=1.76 (0.27 to 3.26) mL/kg/min). Similarly, 6 min walk distance was improved following hybrid (MD=84.78 (31.64 to 138.32) m), centre-based (MD=50.35 (30.15 to 70.56) m) and home-based ExCR (MD=36.77 (12.47 to 61.29) m). Incremental shuttle walk distance did not improve following any ExCR delivery modes. Minnesota living with HF questionnaire improved after centre-based (MD=−10.38 (−14.15 to –6.46)) and home-based ExCR (MD=−8.80 (−13.62 to –4.07)). Kansas City Cardiomyopathy Questionnaire was improved following home-based ExCR (MD=20.61 (4.61 to 36.47)), and Short Form Survey 36 mental component after centre-based ExCR (MD=3.64 (0.30 to 6.14)). HF-related hospitalisation and mortality risks reduced only after centre-based ExCR (OR=0.41 (0.17 to 0.76) and OR=0.42 (0.16 to 0.90), respectively). Mean age of study participants was only associated with changes in VO_2_peak.

**Conclusion:**

ExCR programmes have broader benefits for people with HF and since different delivery modes were comparably effective for improving exercise capacity and HRQoL, the selection of delivery modes should be tailored to individuals’ preferences.

**What is already known about this subject**

- Exercise training is an integral component of heart failure (HF) management and can be administered via several delivery modes. However, the relative effectiveness of different delivery modes remains unclear.

**What does this study add**

- This network meta-analysis is the first to demonstrate the relative effectiveness of different exercise-based cardiac rehabilitation (ExCR) delivery modes on functional, patient-reported and clinical outcomes among people with HF.
- All delivery modes substantially exceeded the minimal clinically important difference (MCID=1 mL/kg/min) for mean changes in VO_2_peak (1.76–3.10 mL/kg/min).
- All delivery modes except technology-enabled ExCR exceeded the 6 min walk distance MCID (30 m).
- All delivery modes exceeded the Minnesota living with HF questionnaire MCID (−5 points), and home-based and technology-enabled modes also exceeded the Kansas City Cardiomyopathy Questionnaire MCID (5.7 points).
- Centre-based ExCR reduced HF-related hospitalisations and HF-related mortality by approximately 60% relative to usual care.

**How might this impact on clinical practice**

- ExCR programmes have broader benefits for people with HF and since different delivery modes are comparably beneficial for exercise capacity and quality of life, selection should be tailored for participants’ preferences and goals, clinical history and risk stratification, and priority outcomes.

## Background

Heart failure (HF) is a major public health problem associated with high mortality and morbidity,^1^ as well as significant reductions in exercise capacity and health-related quality of life (HRQoL).^2^ Clinical guidelines recommend cardiac rehabilitation (CR), a comprehensive intervention comprising exercise and multifactorial education, to achieve and maintain optimal health and prevent further complications for people with HF.^3 4^

Exercise-based CR (ExCR) is recommended as an integral component of comprehensive HF care.^4–6^ ExCR is defined as a supervised or unsupervised exercise training provided to people with cardiac disease in or outside clinical settings and can be provided standalone or as a component of comprehensive CR.^7^ Exercise training improves exercise capacity and quality of life and can reduce hospitalisation and mortality in people with mild-to-moderate chronic HF.^8^ ExTraMATCH II reported HRQoL and exercise capacity were higher after ExCR than no ExCR control.^9^ A Cochrane review reported ExCR improved all-cause and HF-specific hospital admissions and HRQoL.^10^

In pairwise meta-analyses, home-based (HB) ExCR showed significant improvements in exercise capacity and HRQoL over no ExCR control among people with HF.^11 12^ In a meta-analysis of randomised controlled trials (RCTs), a combination of home-based and centre-based (CB) ExCR showed a 9.72 mL/kg/min increase in VO_2_peak over no ExCR control but not in HRQoL (9 RCTs, n=306).^11^ HB ExCR showed greater improvements in VO_2_peak (2.39 mL/kg/min, 18 RCTs, n=1191) and HRQoL (16 RCTs, n=576; standardised mean difference (MD): 0.38) over no ExCR control.^11^ There was no statistically significant difference between HB and CB ExCR in improving exercise capacity and HRQoL.^11 12^

Previous systematic reviews and pairwise meta-analyses reported ExCR has potential health benefits.^9–16^ Since standard meta-analytical procedures can only consider pairwise comparisons, there is limited understanding of how all delivery modes compare. Network meta-analysis (NMA) overcomes this limitation by enabling simultaneous comparisons between more than two treatments.^17^

The aim of this systematic review and NMA was to compare the relative effectiveness of centre, home, technology-enabled (TE) and hybrid ExCR interventions on key HF outcomes (exercise capacity, HRQoL, HF-specific hospitalisation and HF-specific mortality) and to discuss the relative pros and cons of different delivery modes.

## Methods

We conducted and reported this NMA in accordance with the PRISMA extension statement for reporting of systematic reviews incorporating NMA of healthcare interventions^18^ and the PRISMA 2020 statement.^19^

### Search strategy

Six electronic databases (MEDLINE, EMBASE, CINAHL, Cochrane Central Register of Controlled Trials (CENTRAL), Web of Science and PsycINFO) were searched up to 20 June 2021, for studies that combine two key subject areas: HF and exercise. A search strategy including MeSH and free-text terms was developed for MEDLINE and adapted for other databases. The full study protocol, including a detailed search strategy, was registered with PROSPERO before undertaking study selection (ID: CRD42021264709). The search was limited to English-language reports but not restricted by sample size. Reference lists of included studies and relevant systematic reviews and meta-analyses identified by the database search were manually searched for additional studies. Search results were exported to Covidence for duplicate removal, screening, data extraction and quality assessment.

### Eligibility criteria

Eligible studies were RCTs comparing ExCR against usual care (UC) or another ExCR delivery mode among adults (≥18 years) with HF with preserved or reduced ejection fraction. ExCR interventions, either alone or as a component of CR^20^ lasting a minimum of 4 weeks, were included. A 4-week minimum duration aligns with common 30-day postdischarge mortality and hospitalisation outcomes. For this review, ExCR was grouped based on delivery mode into CB, HB, TE and hybrid. Interventions were classified as CB if >50% of programme delivery occurred in traditional clinical settings (eg, hospitals, rehabilitation centres or comparable community facilities), HB if >50% of programme delivery occurred outside traditional clinical settings (eg, clinician home visits, written resources, self-monitoring diaries) without the use of information communication technologies (ICT), and TE if >50% of programme delivery occurred via ICT (eg, video calls, phone calls or text messages) and outside traditional clinical centres. Interventions were classified as hybrid (HY) if they included ≥2 delivery modes, each contributing 20%–50% to programme delivery. Hybrid programmes could use different delivery modes in parallel or sequentially. Eligible comparators were UC (standard medical care including other components of comprehensive CR but excluding exercise training) or ExCR as defined above.

### Outcomes and outcome measures

Studies were included if they reported any of the following ExCR outcomes: exercise capacity, HRQoL, HF-related hospitalisations or HF-related mortality. The primary outcomes were exercise capacity and HRQoL measured on a continuous scale (eg, MD and SD). The secondary outcomes of our analyses were the number of HF-related hospitalisations and HF-related mortality.

### Study selection

Two reviewers independently screened all search results (TKT and RAN) and reviewed full-text papers (TKT and KYA) if the title or abstract identified the eligible population and intervention. Discrepancies were resolved by consensus and/or a third reviewer (JCR).

### Data extraction

Arm-level data were independently extracted into Covidence by two reviewers (TKT and KYA). For each study, data related to study characteristics (intervention and comparator characteristics (eg, delivery mode), sample size, first author, country, year of publication), study population (eg, mean age, gender) and outcomes of interest (as above) were extracted. For studies that had multiple reports, we extracted data for all relevant outcomes without duplication. If outcome data were reported at multiple time points, exercise capacity and HRQoL were extracted at the postintervention time point while HF-related hospitalisation and HF-related mortality were extracted at the longest follow-up time point. If the assessment period for HF-related hospitalisation or mortality were not explicitly reported, we assumed data represented participants entire trial participation period.

### Risk of bias assessment

Two reviewers (TKT and KTK) independently assessed the risk of bias using the Cochrane ‘Risk of Bias 2 (RoB-2)’ tool for RCTs. RoB-2 has five bias domains: bias arising from the randomisation process, bias due to deviations from intended interventions, bias due to missing outcome data, bias in measurement of the outcome and bias in selection of the reported result.^21^ Reviewers assigned a judgement of ‘low risk of bias,’ ‘some concerns,’ or ‘high risk of bias’ for each domain item.^21^ The overall RoB for a study was judged to be at low RoB if all domains were at low RoB, some concerns if at least one domain was at some concerns, and high RoB if at least one domain was at high RoB or judged to have some concerns for multiple domains in a way that substantially lowers confidence in the result.^21^ Discrepancies were resolved through discussion and involving a third author (KYA) when needed.

### Certainty of evidence assessment

The NMA-specific Grading of Recommendations Assessment, Development and Evaluation (GRADE) tool was used to assess the certainty in the evidence^22–25^ based on the following domains: risk of bias, publication bias, imprecision, inconsistency (heterogeneity), incoherence and indirectness.^22 25^ Evidence was rated as ‘high’, ‘moderate’, ‘low’ or ‘very low’.^22 25^ GRADE assessments were performed independently by two reviewers (TKT and KTK). Discrepancies were resolved through discussion.

### Statistical analysis



Bayesian NMA was performed using the gemtc and BUGSnet packages in R. A network graph was generated to provide details of the network geometry. In the network graph, the sizes of the nodes represent the total sample size for each ExCR delivery mode, while line thickness (with a number on it) corresponds to the number of RCTs comparing the ExCR interventions. Model compilation and Markov Chain Monte Carlo simulation were performed to estimate the posterior distributions of model parameters. Continuous and binary outcomes were reported as MDs and OR, respectively, with 95% credible intervals (CrI).

Model convergence was evaluated using trace plots and the Gelman-Rubin-Brooks diagnostics. We used the nma.fit function from the BUGSnet package to identify the best fitting model. This function produced a plot of the leverage values along with the corresponding effective number of parameters, total residual deviance and deviance information criterion. Based on this evaluation, we used the random-effects model to estimate direct, indirect and network effect estimates. Incoherence between direct and indirect effect estimates in closed networks was assessed using the nodesplit method in the gemtc package and the nma.fit and nma.compare functions from the BUGSnet package. Forest plots were used to visualise direct, indirect and network effect estimates.

Furthermore, the surface under the cumulative ranking (SUCRA) function from the dmetar package was used to estimate ranking probabilities for all interventions using a SUCRA curve.^26 27^ The SUCRA score was reported as a percentage, which represents the cumulative probability of a particular intervention being the top-ranking intervention among a set of n interventions. The closer the SUCRA score is to 100%, the higher ranking the intervention in the hierarchy.^26 27^ Ranking probabilities were visualised in SUCRA plots using the nma.rank function in BUGSnet

The relative effectiveness of ExCR interventions could differ across a variety of factors. We performed network meta-regression to determine if trial-level risk of bias, ExCR treatment duration and participant age influenced the magnitude of effect sizes found in the network.

## Results

### Study selection and characteristics of included studies

**Figure 1:**
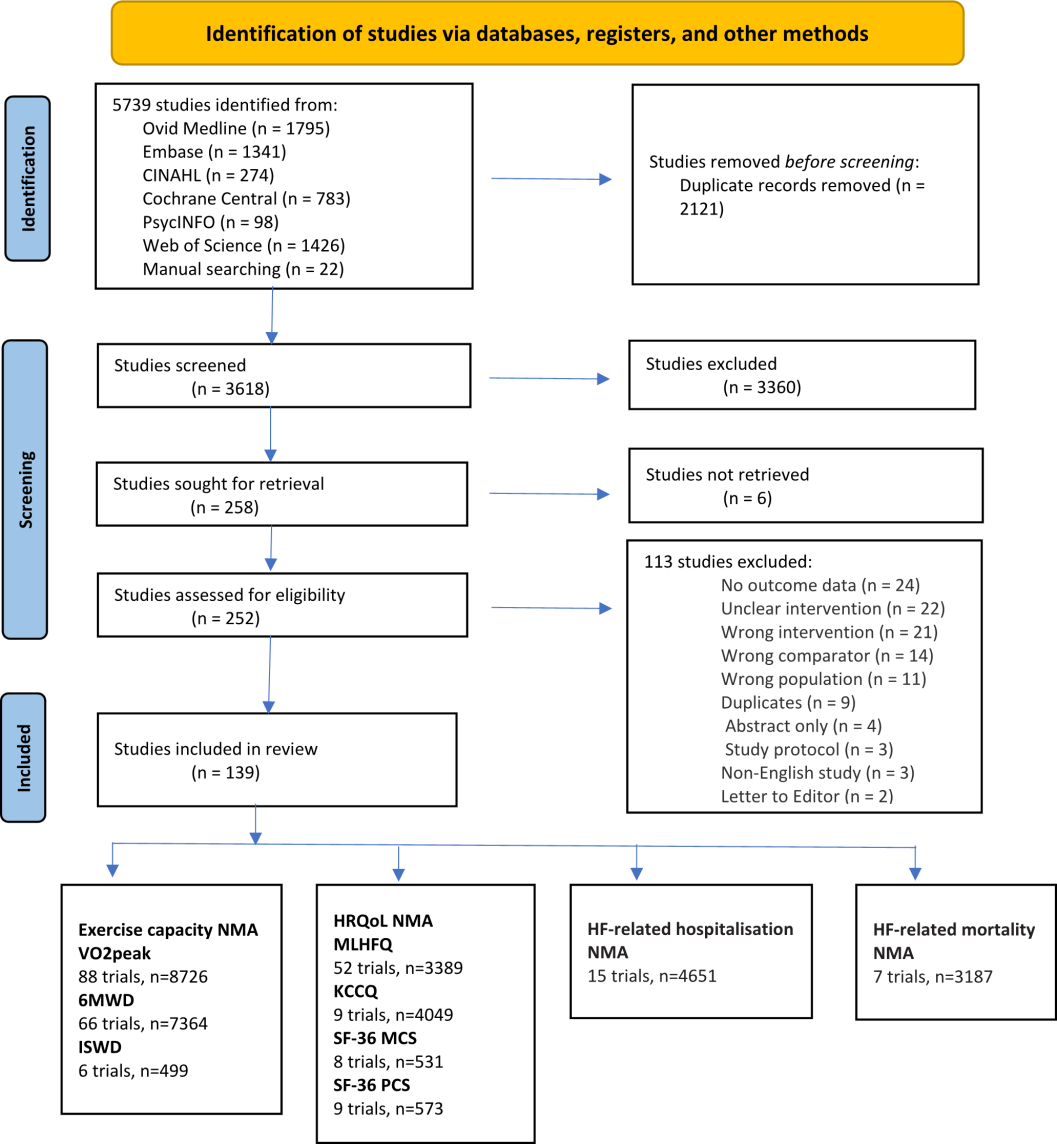
The PRISMA 2020 flow diagram of study selection.^19^ 6MWD, 6-min walk distance; HF, heart failure; HRQoL, health-related quality of life; ISWD, incremental shuttle walk distance; KCCQ, Kansas City Cardiomyopathy Questionnaire; MLHFQ, Minnesota Living with Heart Failure Questionnaire; NMA, network meta-analysis; PRISMA, Preferred Reporting Items for Systematic Reviews and Meta-Analyses; SF-36 MCS, Short Form Survey 36 Mental Component Score; SF-36 PCS, SF 36 Physical Component Score; VO2peak, peak oxygen uptake.

Our systematic search identified 5739 potentially relevant studies, including 22 studies identified from bibliographies of reports of relevant systematic reviews and meta-analysis (figure 1). After full-text screening, we included 139 RCTs, with 18 670 participants conducted between 1996 and 2021. The studies were conducted in 28 countries spread across Europe (eg, UK, Germany, Netherlands, Switzerland), North and South America (eg, USA, Canada, Brazil, Uruguay), Africa (Nigeria), Asia (eg, China, Taiwan) and Australia.

All four ExCR delivery modes were represented. Of the 139 trials, 80 were centred-based vs UC, and 35 were HB vs UC, followed by 9 hybrid vs UC, 7 TE vs UC, 4 centre vs HB, 3 CB vs TE and 1 hybrid vs HB. Detailed information about trial treatments is available in online supplemental file 1, and comparisons are summarised in the network plot figure. A small number of studies reported exercise intensity (n=15) and exercise training compliance (n=18). Aerobic (n=84) and aerobic +resistance (n=27) were the most common training modes, followed by flexibility (n=9), resistance (n=8), aerobic +resistance + flexibility (n=7), aerobic +flexibility (n=2) and resistance +flexibility (n=2). Characteristics of included RCTs^28–166^ are summarised in online supplemental file 1.

10.1136/openhrt-2021-001949.supp1 Supplementary data

The median sample size was 50 participants (range: 10–2331), median participant age was 61.1 years (range: 44–81) and 71.4% of the pooled sample population were male. The median exercise programme duration was 12 weeks (IQR: 12–24 weeks). One study delivered a 10-year programme; however, this comprised three supervised sessions per week for 2 months followed by only two supervised sessions per year.^90^ The median length of study follow-up was 16 weeks (IQR: 12–26 weeks).

Included studies assessed exercise capacity via peak oxygen uptake (VO_2_peak, mL/kg/min) or proxy measures including 6 min walk distance (6MWD, m) and incremental shuttle walk distance (ISWD, m). HRQoL was assessed with the Minnesota Living with Heart Failure Questionnaire (MLHFQ), Kansas City Cardiomyopathy Questionnaire (KCCQ) and Short Survey Form 36 (SF-36) mental and physical components (figure 1). HF-related hospitalisations and HF-related mortalities were reported in absolute numbers.

Of the 139 RCTs, 12 trials reported adverse events that occurred during or immediately after exercise training.^40 43 53 56 74 90 104 110 132 145 156 158^ The reported adverse events were: worsening of HF, hospitalisation due to myocardial infarction, acute coronary syndrome, musculoskeletal injury, shortness of breath, hypoglycaemia, palpitations, angina, arrhythmia, presyncope or syncope, occlusion of peripheral bypass, ectopic heartbeats, hypotension and back pain. No exercise-induced fatal events were reported.

### Risk of bias assessment

**Figure 2:**
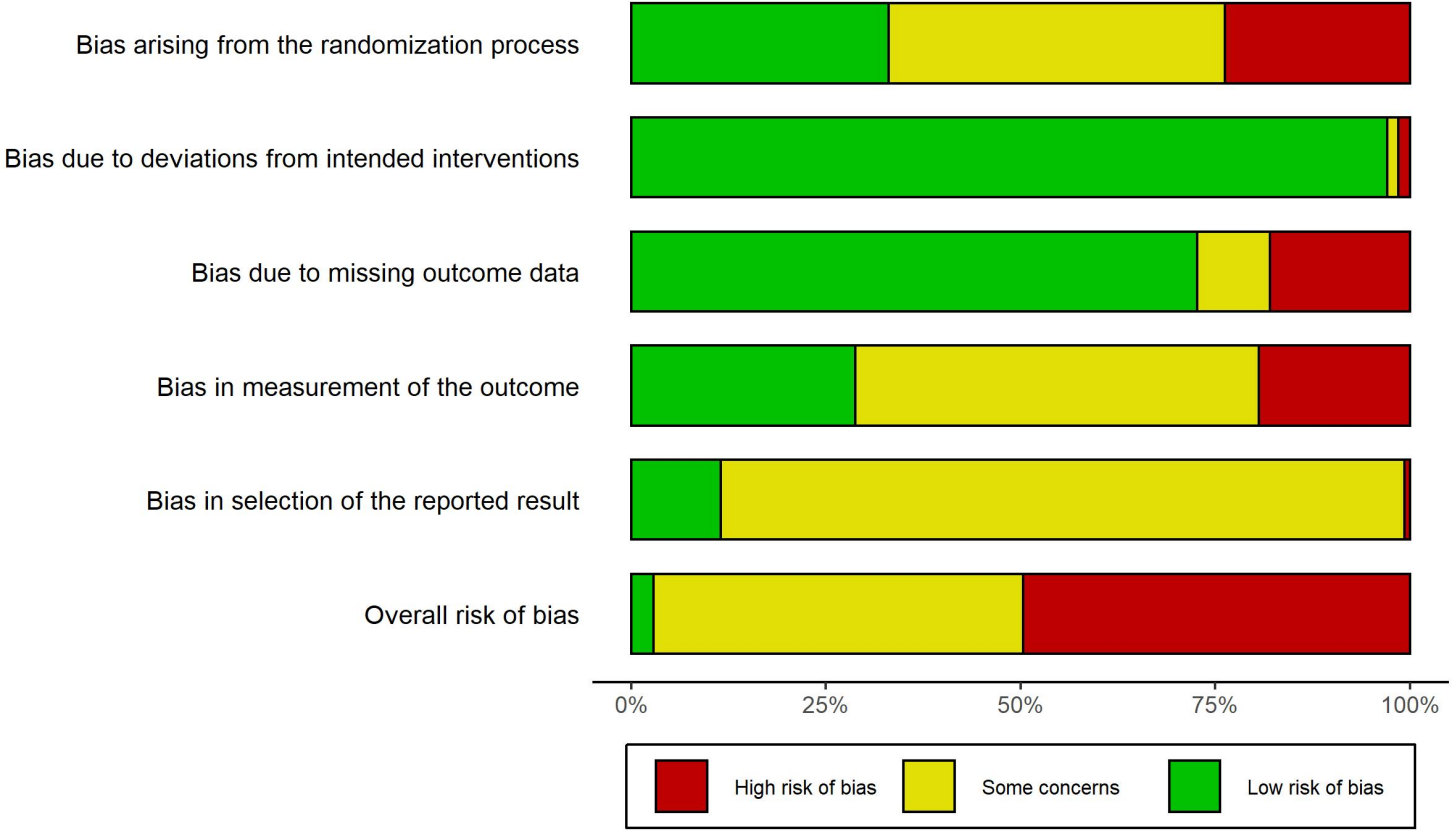
The Cochrane risk of bias graph for the included studies.

Sixty-nine (49.6%) of the 139 RCTs had high overall risk of bias (figure 2); 33 (23.7%) studies had high risk of bias due to the randomization process, 25 (18.8%) due to missing outcome data, 27 (19.4%) due to measurement of the outcome, and one due to selection of the reported result. Two studies^31 120^ had a high risk of bias due to deviations from the intended interventions, where 23 participants crossed over from control to intervention. Of the 139 RCTs, 66 (47.5%) had some concerns about their overall risk of bias: 122 (87.8%) RCTs had some concerns due to the selection of the reported result—studies did not report if they followed a prespecified analysis plan; 72 (51.8%) due to bias in the measurement of the outcome—studies did not report if outcome assessors were blind; and 60 (43.2%) due to the randomization process—studies did not clearly describe allocation concealment. One hundred and thirty-five (97.1%) RCTs had a low risk of bias due to deviations from intended interventions, and 101 (72.7%) due to missing outcome data (figure 2).

### NMA outcomes

**Figure 3:**
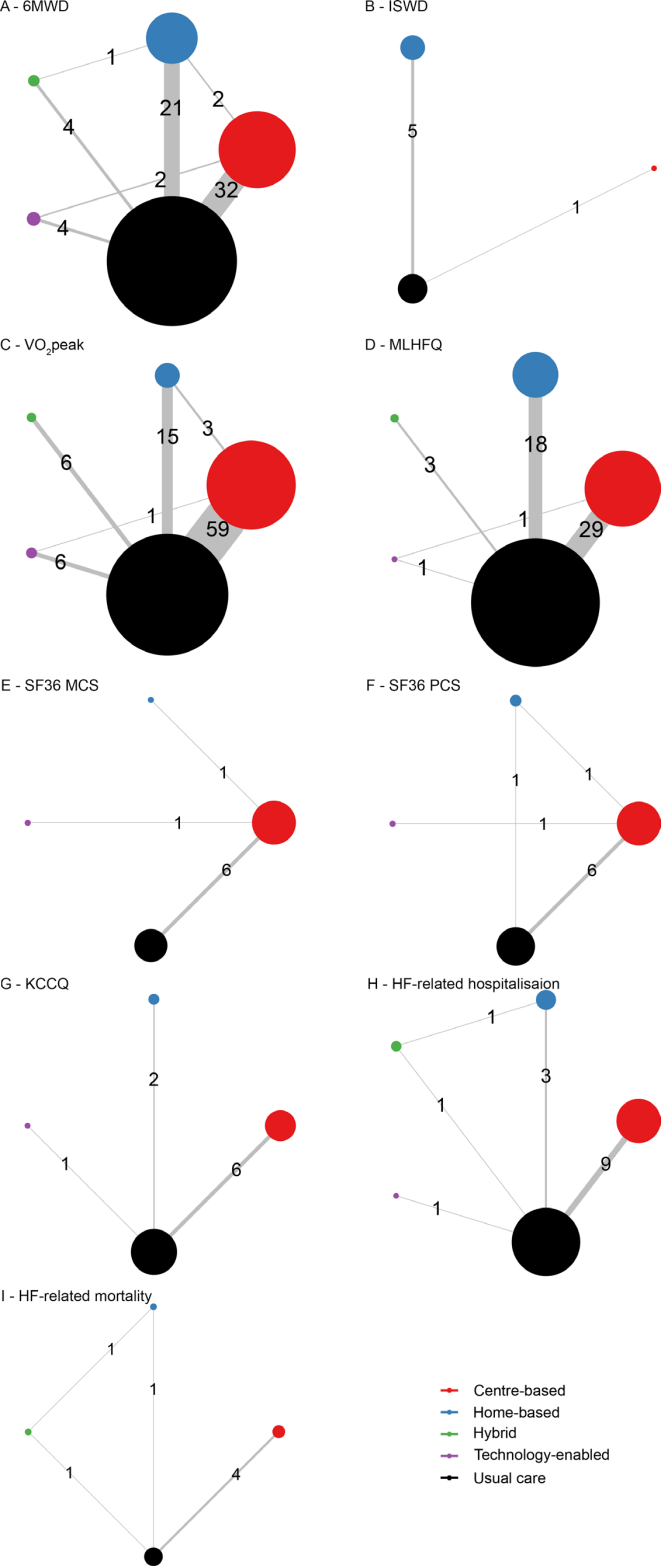
Network geometry for comparisons of treatment effects. 6MWD, 6-min walk distance; HF, heart failure; ISWD, incremental shuttle walk distance; KCCQ, Kansas City Cardiomyopathy Questionnaire; MLHFQ, Minnesota Living with Heart Failure Questionnaire; SF-36 MCS, Short Form Survey 36 Mental Component Score; SF-36 PCS, SF 36 Physical Component Score; VO2peak, peak oxygen uptake.

Network plots of eligible comparisons for all outcome measures are shown in figure 3. GRADE assessments of evidence certainty are presented in online supplemental file 2.

### Exercise capacity

#### Six-min walk distance

Among 66 comparisons of effects on 6MWD, 32 were between CB ExCR and UC followed by 21 HB ExCR and UC (figure 3). Only hybrid, CB and HB ExCR were associated with increases in 6MWD relative to UC (MD (95% CrI)=84.78 (31.64 to 138.32) m: moderate evidence, MD=50.35 (30.15 to 70.56) m: high evidence and MD=36.77 (12.47 to 61.29) m: moderate evidence, respectively). There were no statistically significant differences between delivery modes (online supplemental files 2 and 3).

SUCRA showed that hybrid ExCR had the highest probability of being ranked first (94.6%), followed by CB ExCR (68.8%) and HB ExCR (46.9%) (online supplemental file 4). There was evidence of network heterogeneity (I^2^=97.67%) but not incoherence (p>0.1).

#### Incremental shuttle walk distance

Among six comparisons of effects on ISWD, five were between HB ExCR and UC (figure 3). Neither home or CB programmes improved ISWD compared with UC (HB MD=23.28 (−16.62 to 60.40) m; moderate evidence, and CB MD=9.05 (−70.20 to 88.29) m; low evidence). There was no statistically significant difference between the two ExCR modes (online supplemental files 2 and 3).

Although it did not show statistical significance, SUCRA showed that HB ExCR had the highest probability of being ranked first (76.8%), followed by CB ExCR (48.0%) (online supplemental file 4). There was evidence of network heterogeneity (I^2^=99.05%).

#### Peak oxygen uptake

Among 90 comparisons of effects on VO_2_peak, 59 were between CB ExCR and UC followed by 15 HB ExCR and UC (figure 3). Only CB, HB and TE ExCR were associated with increases in peak oxygen uptake compared with UC (MD=3.10 (2.55 to 3.65) mL/kg/min; high evidence, MD=2.69 (1.67 to 3.70) mL/kg/min; moderate evidence and MD=1.76 (0.26, 3.26) mL/kg/min: low evidence, respectively). There were no statistically significant differences between delivery modes (online supplemental files 2 and 3).

SUCRA showed that CB ExCR had the highest probability of being ranked first (90.5%), followed by HB ExCR (71.8%) and hybrid ExCR (44.1%) (online supplemental file 4). There was evidence of network heterogeneity (I^2^=94.59%) but not incoherence (p>0.1).

### Health-related quality of life

#### MLHFQ score

Among 52 comparisons of effects on MLHFQ, 29 were between CB ExCR and UC followed by 18 HB ExCR and UC (figure 3). Only centre and HB ExCR showed significant decreases in MLHFQ score compared with UC (MD=−10.38 (−14.15 to –6.46); high evidence, and MD=−8.80 (−13.62 to –4.07); low evidence, respectively). There were no statistically significant differences between delivery modes (online supplemental files 2 and 3).

SUCRA showed that TE ExCR had the highest probability of being ranked first (70.6%), followed by CB ExCR (66.6%) and hybrid ExCR (56.6%) (online supplemental file 4). There was evidence of network heterogeneity (I^2^=98.05%) but not incoherence (p>0.1).

#### SF-36 mental component summary score

Among eight comparisons of effects on the SF-36 mental component summary score, six were between CB ExCR and UC (figure 3). Only CB delivery was associated with a statistically significant increase relative to UC (MD (95% CrI)=3.64 (0.30 to 6.14); moderate evidence). There were no statistically significant differences between the CB, HB or TE delivery modes (online supplemental files 2 and 3).

SUCRA showed that CB ExCR had the highest probability of being ranked first (74.7%), followed by TE (70.4%) and HB ExCR (40.2%) (online supplemental file 4). There was evidence of network heterogeneity (I^2^=83.4%).

#### SF-36 physical component summary score

Among nine comparisons of effects on SF-36 physical component summary score, six were between CB ExCR and UC (figure 3). No delivery mode improved the SF-36 physical component summary score compared with UC (CB MD=3.24 (−0.37 to 7.35); moderate evidence, HB MD=3.28 (−3.63 to 10.74); high evidence) and TE MD=3.59 (−5.38 to 13.21); moderate evidence). There were no statistically significant differences between the three modes (online supplemental files 2 and 3).

Although it did not show statistical significance, SUCRA showed that CB ExCR had the highest probability of being ranked first (63.8%), followed by TE (62.6%) and HB ExCR (60.4%) (online supplemental file 4). There was evidence of network heterogeneity (I^2^=98.18%) but not incoherence (p>0.1).

#### KCCQ score

Among nine comparisons of effects on KCCQ, six were between CB ExCR and UC (figure 3). Only HB ExCR was associated with a significant increase in KCCQ relative to UC (MD=20.61 (4.61 to 36.47); moderate evidence). There were no statistically significant differences between delivery modes (online supplemental files 2 and 3).

SUCRA showed that HB ExCR had the highest probability of being ranked first (95.6%), followed by TE ExCR (56.9%) and CB ExCR (41.5%) (online supplemental file 4). There was evidence of network heterogeneity (I^2^=98.77%).

### HF-related hospitalisation

Among 15 comparisons of effects on HF-related hospitalisation, nine were between CB ExCR and UC, and included relatively short observation periods (4–60 weeks) except for one study with a 520-week treatment period^90^ (figure 3). CB ExCR was the only delivery mode associated with lower HF-related hospitalisation risk (OR=0.41 (95% CrI 0.17 to 0.76): high evidence), and HF-related hospitalisation risk did not differ between ExCR delivery modes (online supplemental files 2 and 3).

SUCRA showed that hybrid ExCR had the highest probability of being ranked first (75.2%), followed by HB ExCR (71.7%) and CB ExCR (66.2%) (online supplemental file 4). There was evidence of network heterogeneity (I^2^=87.81%) but not incoherence (p>0.1).

### HF-related mortality

Only seven comparisons assessed effects on HF-related mortality; four were between CB ExCR and UC, and included relatively short observation periods (12–60 weeks) except the one study with a 520-week treatment period^90^ (figure 3). Similar to HF-related hospitalisation, CB ExCR was the only delivery mode associated with lower HF-related mortality risk (OR=0.42 (95% CrI 0.16 to 0.90): moderate evidence), and effects did not differ between ExCR delivery modes (online supplemental files 2 and 3).

SUCRA showed that hybrid ExCR had the highest probability of being ranked first (88.9%), followed by CB ExCR (56.9%) and HB ExCR (45.0%; online supplemental file 4). There was neither network heterogeneity (I^2^=0) nor incoherence (p>0.1).

### Network meta-regression

Mean age of study participants was significantly associated with changes in VO_2_peak (β (95% CrI)=−1.41 (–2.37 to –0.46)), but not with other outcomes. After controlling for age, only CB, TE and HB ExCR were associated with significant increases in VO_2_peak relative to UC (MD=3.22 (2.69 to 3.75) mL/kg/min, MD=1.90 (0.46 to 3.33) mL/kg/min and MD=2.52 (1.55 to 3.50) mL/kg/min, respectively). Risk of bias and exercise programme duration were not significantly associated with any outcomes (results not presented).

## Discussion

This NMA is the first to demonstrate the relative effectiveness of different ExCR delivery modes on functional, patient-reported and clinical outcomes among people with HF. While the quality of evidence and number of studies included in each comparison varied markedly the overall results across delivery modes are consistent with previous research evaluating the benefits of ExCR among people with HF.^167–171^

As the mainstay approach in many countries, CB delivery has been studied extensively and was associated with improvements in at least one measure of exercise capacity and HRQoL as well as HF-related hospitalisation and mortality.^16 20 172 173^ HB delivery was the next most widely studied mode and, consistent with previous pairwise meta-analyses, it was associated with improvements in exercise capacity and HRQoL but not hospitalisation or mortality risks.^11 12^ Neither centre nor HB delivery modes improved ISWD. Only six studies, with relatively small numbers of participants (ranges from 33 to 65) and a high risk of bias, evaluated the effect of centre and HB ExCR on ISWDe. Effect estimates of comparisons involving few studies with a small number of participants and low to moderate evidence suggest this should be interpreted with caution pending further research. While few published studies have evaluated TE or hybrid^11^ delivery modes among people with HF, both were associated with improvements in exercise capacity. Neither TE nor hybrid delivery improved HF-related hospitalisation or mortality risk; however, small numbers of studies mean it may be too soon to draw definitive conclusions about the effects of hybrid and TE delivery on clinical outcomes.

While not all delivery modes were effective for all outcome measures, it is important to note we found no evidence of differential effectiveness between delivery modes. Small numbers of comparisons and low to moderate evidence suggest this should be interpreted with caution pending further research, but comparable outcomes between delivery modes are promising given the impact of accessibility barriers on rates of participation in CB programmes (eg, transportation problem and travel costs, distance to rehabilitation centres and rehabilitation costs).^174^ Effective HB, TE or hybrid delivery modes may help to increase uptake and adherence by enabling people to undertake ExCR in more accessible locations. While these delivery modes did not improve key clinical endpoints compared with UC, mean changes in VO_2_peak (1.76–3.10 mL/kg/min) substantially exceeded the clinically important difference associated with reduced mortality risk (1 mL/kg/min).^169 170^ All delivery modes except TE ExCR exceeded minimal clinically important difference (MCID)=30 m of 6MWD,^167 168^ and HB and TE modes exceeded MCID=5.7 points of KCCQ.^171^ Similarly, all delivery modes exceeded MCID=−5 points of MLHFQ.^175–177^

Implementing a range of different ExCR modes in clinical practice—including hybrid options—could be important to maximise uptake rates and adherence by meeting a wide range of participant needs and preferences, and safety concerns.^11^ Hybrid ExCR could be done in any order/sequence to form a cohesive and comprehensive CR programme. This may be particularly beneficial for people who experience challenges accessing CB programmes, but risk stratification indicates a need for direct supervision by a healthcare professional. For instance, initial CB sessions could be undertaken to manage physical and psychosocial risks, increase participation in group education sessions, and tailor the exercise regimen based on direct observation. When appropriate, subsequent transition into TE^178^ could aid adherence by reducing accessibility challenges while preserving a level of supervision and monitoring.

In addition to the relative effect estimates, we also reported cumulative ranking probabilities which support to assist decision making by identifying the likelihood of a particular treatment would be best for a specific outcome.^27^ This may be most useful when the rankings of cumulative probabilities and effect estimates align—as was the case for 6MWD in this review (hybrid ExCR effect estimate=84.78 m, cumulative probability of ranking first=94.6%; CB ExCR effect estimate=50.35 m, cumulative probability of ranking second=68.8%; HB ExCR effect estimate=36.77 m, cumulative probability of ranking third=46.9%). However, use of rankings to inform decision making requires some caution because they do not account for the quality of underpinning evidence, magnitudes of differences between individual treatments, or the possibility differences between treatments may be explained by chance. Moreover, as ranking probabilities relate to a single outcome they do not consider the importance other relevant benefits, harms or pragmatic factors such as cost and complexity.^27^ Therefore, the selection of ExCR delivery modes should consider a wide range of factors in addition to probability rankings, and the most desirable option(s) may vary between individuals and across healthcare contexts.

This NMA was not without limitations. Few studies with relatively small numbers of participants evaluated TE and hybrid ExCR, therefore, effect estimates of comparisons involving these delivery modes were imprecise. Second, the methods of included studies were not well described, and most studies were judged to have some concerns of risk of bias. Specifically, few studies adequately described the randomisation process (allocation concealment), outcome assessment (outcome assessor blinding) or whether analyses followed a prespecified plan (selection of reported results). Although overall risk of bias was not associated with outcome effects in the network meta-regression, several studies were judged to have high risk of bias. In addition, the results of this NMA could be biased for numerous causes including heterogeneity in study population (eg, gender and age), exercise regimen and intensity of the training, compliance to training. Finally, interpretation of effect estimates on HF-related hospitalisation and mortality are impacted by a very broad range of follow-up periods, and a lack of explicit reporting of the follow-up period in some studies.

## Conclusion

ExCR programmes improve functional capacity, quality of life and/or clinical outcomes compared with UC, regardless of whether they are delivered in clinical centres, at home, via digital technologies or a combination of these. ExCR services should consider offering different delivery modes to meet a wider range of participant needs and preferences, and mode selection should consider factors such as individual preferences and goals, clinical history and risk stratification, and priority outcomes.

## Data Availability

All data relevant to the study are included in the article or uploaded as online supplemental information.
