# Supplementary material for: Effects of exercise-based cardiac rehabilitation delivery modes on exercise capacity and health-related quality of life in heart failure: a systematic review and network meta-analysis
Source: Open Heart. 2022 Jun 9;9(1):e001949. doi: 10.1136/openhrt-2021-001949 (PMC9185675; doi:10.1136/openhrt-2021-001949)
Supplement: Supplementary data [file openhrt-2021-001949supp001.pdf]

Supplementary data belonging to:

Effects of exercise-based cardiac rehabilitation delivery modes on exercise capacity and health-related quality of life in heart failure: a systematic review and network meta-analysis

Teketo Kassaw Tegegne, Jonathan Charles Rawstorn, Rebecca Amy Nourse, Kelemu Tilahun Kibret, Kedir Yimam Ahmed, Ralph Maddison

Abbreviations:

6MWD – Six-minute walk distance, ISWD – Incremental shuttle walk distance, VO2peak – Peak oxygen uptake, MLHFQ – Minnesota Living with Heart Failure Questionnaire, SF-36 MCS – Short Form Survey 36 mental component score, SF-36 PCS – Short Form Survey 36 physical component score, KCCQ – Kansas City Cardiomyopathy Questionnaire, HF – Heart failure

Tegegne TK, *et al. Open Heart* 2022; 9:e001949. doi: 10.1136/openhrt-2021-001949

Supplement 1 Characteristics of the included studies

Study	Country of study	Exercise program duration (weeks)	Follow-up duration (weeks)	Sample size	ExCR delivery modes	NYHA class	HF type
Ajiboye 2015 [87]	Nigeria	12	12	51	CB, UC	NYHA II - III	Not reported
Aksoy 2015 [88]	Turkey	10	10	45	CB, UC	NYHA II - III	Not reported
Andryukhin 2010 [146]	Russia	24	24	85	HB, UC	NYHA I - III	HFpEF
Austin 2005 [133]	UK	24	24	200	CB, UC	NYHA II - III	Not reported
Azhar 2020 [161]	USA	12	12	11	CB, UC	NYHA II - III	HFpEF
Barrow 2007 [131]	UK	16	16	65	CB, UC	NYHA II - III	Not reported
Beckers 2010 [162]	Netherlands	12	52	53	HB, CB, UC	NYHA II - III	Not reported
Belardinelli 1999 [145]	Italy	60	60	99	CB, UC	NYHA I - IV	Not reported
Belardinelli 2005 [91]	Italy	8	8	59	CB, UC	NYHA II - III	Not reported
Belardinelli 2006 [89]	Italy	8	78	52	CB, UC	NYHA II - III	Not reported
Belardinelli 2012 [90]	Italy	520	520	123	CB, UC	NYHA II - III	Not reported
Bernocchi 2018 [152]	Italy	16	26	112	HB, UC	NYHA II - IV	Not reported
Blumenthal 2012 [140]	USA, Canada & France	12	208	2,322	CB, UC	NYHA I - IV	Not reported
Borland 2014 [30]	Sweden	12	12	48	UC, HB	NYHA II - III	Not reported
Brubaker 2009 [142]	USA	16	16	59	CB, UC	NYHA II - III	HFrEF
Brubaker 2020 [28]	USA	16	16	116	UC, CB	NYHA II - III	HFrEF
Butterfield 2008 [92]	Australia	12	12	19	HB, UC	Not reported	Not reported
Chen 2018c [93]	China	24	24	62	CB, UC	NYHA II - IV	Not reported

Study	Country of study	Exercise program duration (weeks)	Follow-up duration (weeks)	Sample size	ExCR delivery modes	NYHA class	HF type
Chen 2018t [94]	Taiwan	12	12	37	HB, UC	NYHA I - III	HFrEF
Chen 2018ta [135]	Taiwan	12	12	80	HB, UC	NYHA I - II	Not reported
Chien 2011 [95]	Taiwan	8	8	51	HB, UC	NYHA I - III	Not reported
Chou 2019 [96]	Taiwan	12	12	34	CB, UC	NYHA II - III	Not reported
Cider 2003 [138]	Sweden	8	8	25	CB, UC	NYHA II - III	Not reported
Corvera-Tindel 2004 [97]	USA	12	12	79	HB, UC	NYHA II - IV	Not reported
Dalal 2019 [98]	UK	12	52	216	HB, UC	NYHA I - III	HFrEF
Daskapan 2005 [99]	Turkey	12	12	22	HB, CB	NYHA II - III	Not reported
Davidson 2010 [141]	Australia	12	12	105	HY, UC	NYHA I - III	Not reported
deMeirelles 2014 [137]	Brazil	24	26	30	CB, UC	NYHA II - III	Not reported
deMelloFranco 2006 [63]	Brazil	16	16	29	CB, UC	NYHA II - III	Not reported
Dracup 2007 [67]	USA	52	52	173	HB, UC	NYHA II - IV	Not reported
Du 2018 [57]	Australia	24	26	132	HB, UC	NYHA II - III	Not reported
Dubach 1997 [66]	Switzerland	8	8	25	CB, UC	NYHA II - III	Not reported
Dziekan 1998 [65]	Switzerland	8	8	20	CB, UC	NYHA II - III	Not reported
Edelmann 2011 [74]	Germany	12	12	64	CB, UC	NYHA II - III	HFpEF
Ellis 2020 [100]	Australia	8	8	47	CB, UC	NYHA I - III	Not reported
Erbs 2010 [62]	Germany	12	12	37	HB, UC	NYHA III	Not reported
Evangelista 2006 [69]	USA	24	26	99	HB, UC	NYHA II - IV	Not reported
Evans 2010 [71]	UK	7	7	57	HB, UC	NYHA II - IV	Not reported

Tegegne TK, *et al. Open Heart* 2022; 9:e001949. doi: 10.1136/openhrt-2021-001949

Study	Country of study	Exercise program duration (weeks)	Follow-up duration (weeks)	Sample size	ExCR delivery modes	NYHA class	HF type
Flynn 2009 [155]	USA, Canada & France	12	208	2,331	CB, UC	NYHA I - IV	Not reported
Fraga 2007 [147]	Brazil	16	16	27	CB, UC	NYHA II - III	Not reported
Fu 2013 [101]	Taiwan	12	12	45	CB, UC	NYHA II - III	Not reported
Gary 2004 [44]	USA	12	12	32	HB, UC	NYHA II - III	Not reported
Gary 2007 [68]	USA	12	12	23	HB, UC	NYHA II - III	Not reported
Gary 2010 [136]	USA	12	24	37	HB, UC	NYHA II - III	Not reported
Gary 2012 [157]	USA	12	12	24	HB, UC	NYHA II - III	Not reported
Giannuzzi 2003 [144]	Italy	24	26	90	HB, UC	NYHA II - III	Not reported
Gielen 2003 [102]	Switzerland	24	26	20	HB, UC	NYHA II - III	Not reported
Glowczynska 2021 [154]	Poland	9	9	782	TE, UC	NYHA I - III	Not reported
Guazzi 2004 [36]	Italy	8	16	31	CB, UC	NYHA I - III	Not reported
Hambrecht 2000 [39]	Germany	24	26	73	HB, UC	NYHA I - III	Not reported
Hambrecht 2005 [37]	Germany	24	26	18	HB, UC	NYHA II - III	Not reported
Hasanpour-Dehkordi 2020 [165]	Iran	24	24	52	CB, UC	NYHA II - III	Not reported
Hollriegel 2016 [53]	Germany	52	52	37	HB, UC	NYHA III	Not reported
Hwang 2017 [38]	Australia	12	24	53	CB, TE	NYHA I - III	HFpEF & HFrEF
Jaarsma 2021 [128]	Sweden, Italy, Israel, Netherlands, Germany & USA	12	52	605	HB, UC	NYHA I - IV	Not reported
Jolly 2009 [134]	UK	24	52	169	HB, UC	NYHA I - III	Not reported
Jones 2014 [132]	USA, Canada & France	12	208	90	CB, UC	NYHA I - IV	Not reported

Study	Country of study	Exercise program duration (weeks)	Follow-up duration (weeks)	Sample size	ExCR delivery modes	NYHA class	HF type
Jonsdottir 2006 [47]	Iceland	20	52	43	CB, UC	NYHA II - III	Not reported
Kaltsatou 2014 [103]	Greek	32	34	51	CB, UC	NYHA II - III	Not reported
Karapolat 2009 [70]	Turkey	8	8	68	HB, CB	NYHA II - III	Not reported
Keteyian 1996 [35]	USA	24	24	29	CB, UC	NYHA II - III	Not reported
Kiilavuori 1999 [81]	Finland	24	26	27	HY, UC	NYHA II - III	Not reported
Kitzman 2010 [41]	USA	16	16	53	CB, UC	NYHA II - III	HFpEF
Kitzman 2013 [40]	USA	16	16	63	CB, UC	NYHA II - III	HFpEF
Kitzman 2016 [43]	USA	20	20	100	CB, UC	NYHA II - III	HFpEF
Kitzman 2021 [42]	USA	12	26	349	CB, UC	NYHA II - IV	Not reported
Klecha 2007 [82]	Poland	24	26	50	CB, UC	NYHA II - III	Not reported
Klocek 2005 [85]	Poland	24	26	42	CB, UC	NYHA II - III	Not reported
Koukouvou 2004 [79]	Greek	24	26	26	CB, UC	NYHA II - III	Not reported
Kulcu 2007 [75]	Turkey	8	8	44	CB, UC	NYHA II - III	Not reported
Lang 2018 [159]	UK	12	26	50	HB, UC	NYHA II - III	HFpEF
Lans 2018 [150]	Sweden	12	52	22	HB, CB	NYHA II - III	Not reported
Maiorana 2011 [34]	Australia	12	12	36	CB, UC	NYHA I - III	Not reported
Maldonado-Martin 2017 [130]	USA	16	16	47	CB, UC	NYHA II - III	HFpEF
Mandic 2009 [104]	Canada	12	12	42	CB, UC	NYHA I - III	Not reported
McKelvie 2002 [60]	Canada	52	52	181	HB, UC	NYHA I - III	Not reported
Mezzani 2013 [105]	Italy	12	12	30	HB, UC	NYHA II - III	Not reported

Tegegne TK, *et al. Open Heart* 2022; 9:e001949. doi: 10.1136/openhrt-2021-001949

Study	Country of study	Exercise program duration (weeks)	Follow-up duration (weeks)	Sample size	ExCR delivery modes	NYHA class	HF type
Mudge 2018 [31]	Australia	12	52	278	HB, HY	NYHA I - IV	HFpEF & HFrEF
Mueller 2007 [106]	Switzerland	4	322	50	CB, UC	Not reported	Not reported
Mueller 2021 [56]	Germany, Belgium & Norway	52	52	176	TE, UC	NYHA II - III	HFpEF
Muller 2009 [107]	Switzerland	4	322	16	CB, UC	NYHA II - III	Not reported
Myers 2001 [76]	Switzerland	8	8	24	CB, UC	NYHA II - III	Not reported
Myers 2007 [108]	Switzerland	8	8	24	CB, UC	NYHA II - III	Not reported
Myers 2012 [73]	Switzerland	8	8	50	CB, UC	NYHA II - III	Not reported
Nilsson 2008 [78]	Norway	16	16	80	CB, UC	NYHA II - III	Not reported
Nilsson 2010 [109]	Norway	16	16	78	CB, UC	NYHA II - III	Not reported
Nolte 2015 [110]	Germany	12	12	64	CB, UC	NYHA II - III	HFpEF
Norman 2020 [151]	USA	76	78	204	CB, UC	NYHA I - IV	Not reported
O'Connor 2009 [158]	USA, Canada & France	12	208	2,331	CB, UC	NYHA I - IV	Not reported
Oka 2000 [111]	USA	12	12	40	HB, UC	NYHA II - III	Not reported
Passino 2006 [112]	Italy	36	39	85	HB, UC	NYHA I - III	Not reported
Passino 2008 [58]	Italy	36	39	90	HB, UC	NYHA I - III	Not reported
Patwala 2009 [113]	UK	12	12	50	CB, UC	NYHA II - IV	Not reported
Peng 2018 [84]	China	8	16	98	TE, UC	NYHA I - III	Not reported
Piotrowicz 2010 [153]	Poland	8	8	131	CB, TE	NYHA II - III	Not reported
Piotrowicz 2015 [33]	Poland	8	8	107	TE, UC	NYHA II - III	Not reported

Study	Country of study	Exercise program duration (weeks)	Follow-up duration (weeks)	Sample size	ExCR delivery modes	NYHA class	HF type
Piotrowicz 2015b [166]	Poland	8	8	131	CB, TE	NYHA II - III	Not reported
Piotrowicz 2016 [139]	Poland	8	8	69	TE, UC	NYHA II - III	Not reported
Piotrowicz 2020 [163]	Poland	9	104	850	TE, UC	NYHA I - III	Not reported
Pourhabib 2018 [59]	Iran	12	12	53	CB, UC	NYHA II - III	Not reported
Pozehl 2010 [32]	USA	12	12	42	CB, UC	NYHA II - III	Not reported
Pullen 2008 [114]	USA	8	8	19	HY, UC	NYHA I - III	Not reported
Pullen 2010 [29]	USA	8	8	40	CB, UC	NYHA I - III	Not reported
Quittan 1999 [54]	Austria	12	12	25	CB, UC	NYHA I - III	Not reported
Redwine 2019 [115]	USA	16	16	70	CB, UC	Not reported	HFpEF & HFrEF
Reeves 2017 [156]	USA	12	26	27	CB, UC	Not reported	Not reported
Ricca-Mallada 2017 [51]	Uruguay	24	24	34	CB, UC	NYHA I - III	Not reported
Roveda 2003 [55]	Brazil	16	16	16	CB, UC	NYHA II - III	Not reported
Sabelis 2004a [116]	Netherlands	26	26	29	HY, UC	NYHA II - III	Not reported
Sabelis 2004b [117]	Netherlands	26	26	61	HY, UC	NYHA II - III	Not reported
Sadek 2020 [118]	Lebanon	12	12	20	CB, UC	NYHA II - III	Not reported
Safiyari-Hafizi 2016 [50]	Canada	12	12	40	HB, UC	NYHA I - III	Not reported
Santa-Clara 2019 [80]	Portugal	24	26	37	CB, UC	NYHA II - IV	Not reported
Santos 2010 [119]	Brazil	16	16	23	CB, UC	NYHA I - III	Not reported

Tegegne TK, et al. Open Heart 2022; 9:e001949. doi: 10.1136/openhrt-2021-001949

Study	Country of study	Exercise program duration (weeks)	Follow-up duration (weeks)	Sample size	ExCR delivery modes	NYHA class	HF type
Senden 2005 [120]	Netherlands	26	26	61	HY, UC	NYHA II - III	Not reported
Servantes 2012 [121]	Brazil	12	12	45	HB, UC	NYHA II - III	Not reported
Servantes 2018 [48]	Brazil	12	12	37	CB, UC	NYHA II - III	Not reported
Shoemaker 2017 [86]	USA	12	39	10	HB, UC	NYHA II - III	Not reported
Silva 2002 [164]	Brazil	12	12	24	CB, UC	NYHA I - III	Not reported
Smart 2012 [49]	Australia	16	16	25	CB, UC	NYHA I - II	HFpEF
Smolis-Bak 2015 [61]	Poland	12	52	52	TE, UC	NYHA III	Not reported
Smolis-Bak 2017 [46]	Poland	24	78	84	CB, UC	NYHA III	Not reported
Spee 2016 [143]	Netherlands	12	12	26	CB, UC	NYHA I - III	Not reported
Spee 2020 [122]	Netherlands	12	12	24	CB, UC	NYHA II - III	Not reported
Sturm 1999 [148]	Austria	12	12	26	CB, UC	NYHA II - III	Not reported
Tyni-Lenne 1996 [52]	Sweden	12	8	21	CB, UC	NYHA II - III	Not reported
Tyni-Lenne 2001 [83]	Sweden	8	8	24	CB, UC	NYHA II - III	Not reported
Vordos 2017 [77]	Greek	12	12	33	CB, UC	NYHA I - II	Not reported
Wielenga 1999 [72]	Netherlands	12	12	67	CB, UC	NYHA II - III	Not reported
Willenheimer 1998 [160]	Sweden	16	16	49	CB, UC	NYHA I - III	Not reported
Willenheimer 2001 [123]	Sweden	16	43	37	CB, UC	NYHA I - IV	Not reported
Williams 2007 [124]	Australia	12	12	13	CB, UC	NYHA II - III	Not reported
Witham 2005 [126]	UK	24	26	82	HY, UC	NYHA II - III	Not reported
Witham 2007 [125]	UK	24	82	82	HY, UC	NYHA II - III	Not reported

Study	Country of study	Exercise program duration (weeks)	Follow-up duration (weeks)	Sample size	ExCR delivery modes	NYHA class	HF type
Xueyu 2017 [127]	China	12	12	78	HB, UC	NYHA II - III	Not reported
Yeh 2004 [45]	USA	12	12	30	CB, UC	NYHA I - IV	Not reported
Yeh 2008 [64]	USA	12	12	30	HY, UC	NYHA I - IV	Not reported
Yeh 2011 [149]	USA	12	12	100	CB, UC	NYHA I - III	Not reported
Zeitler 2015 [129]	USA, Canada & France	12	208	1,213	CB, UC	NYHA II – IV	Not reported

CB – Centre-based ExCR, HB – Home-based ExCR, HY – Hybrid ExCR, TE – Technology-enabled ExCR and UC – Usual care

HFpEF – Heart Failure with preserved Ejection Fraction, and HFrEF – Heart Failure with reduced Ejection Fraction

NYHA – New York Heart Association Functional Classification

Supplement 2 GRADE assessments

Comparison	Within-study bias	Reporting bias	Indirectness	Imprecision	Heterogeneity	Incoherence	Confidence rating
**6MWD**							
CB vs HB	Some concerns	No concerns	No concerns	No concerns	No concerns	No concerns	High
CB vs TE	Some concerns	No concerns	No concerns	No concerns	No concerns	No concerns	High
CB vs UC	Some concerns	No concerns	No concerns	No concerns	No concerns	No concerns	High
HB vs TE	Some concerns	No concerns	No concerns	No concerns	No concerns	No concerns	High
HB vs HY	Major concerns	No concerns	Some concerns	No concerns	No concerns	No concerns	Moderate
HB vs UC	Some concerns	No concerns	No concerns	No concerns	Major concerns	No concerns	Moderate
HY vs UC	Major concerns	Some concerns	Some concerns	No concerns	No concerns	No concerns	Moderate
TE vs UC	Major concerns	Some concerns	No concerns	Some concerns	No concerns	No concerns	Moderate
CB vs HY	Major concerns	Some concerns	No concerns	Some concerns	No concerns	No concerns	Moderate
HY vs TE	Major concerns	Some concerns	No concerns	Some concerns	No concerns	No concerns	Moderate
**ISWD**							
HB vs UC	Some concerns	No concerns	No concerns	No concerns	Major concerns	No concerns	Moderate
CB vs UC	Major concerns	Some concerns	No concerns	Major concerns	No concerns	No concerns	Low
CB vs HB	Major concerns	Some concerns	No concerns	Major concerns	No concerns	No concerns	Low
**VO_2peak**							
CB vs UC	Some concerns	No concerns	Some concerns	No concerns	No concerns	No concerns	High
CB vs HY	Some concerns	No concerns	Some concerns	No concerns	No concerns	No concerns	High
CB vs HB	Major concerns	No concerns	Some concerns	Some concerns	No concerns	No concerns	Moderate
CB vs TE	Major concerns	No concerns	Some concerns	Some concerns	No concerns	No concerns	Moderate
HB vs UC	Major concerns	No concerns	Some concerns	No concerns	No concerns	No concerns	Moderate

HY vs UC	Major concerns	Some concerns	No concerns	Some concerns	No concerns	No concerns	Moderate
HB vs TE	Major concerns	No concerns	Some concerns	Some concerns	No concerns	No concerns	Moderate
TE vs UC	Major concerns	Some concerns	Some concerns	No concerns	Major concerns	No concerns	Low
HB vs HY	Major concerns	Some concerns	Some concerns	Major concerns	No concerns	No concerns	Low
HY vs TE	Major concerns	Some concerns	Some concerns	Major concerns	No concerns	No concerns	Low
**MLHFQ**							
CB vs UC	Some concerns	No concerns	No concerns	No concerns	No concerns	No concerns	High
HY vs UC	Some concerns	Some concerns	No concerns	No concerns	No concerns	No concerns	High
TE vs UC	Some concerns	No concerns	No concerns	Some concerns	No concerns	No concerns	High
CB vs TE	Some concerns	No concerns	Some concerns	Major concerns	No concerns	No concerns	Moderate
CB vs HB	Some concerns	No concerns	Some concerns	No concerns	Major concerns	No concerns	Moderate
CB vs HY	Some concerns	Some concerns	No concerns	Major concerns	No concerns	No concerns	Moderate
HB vs HY	Some concerns	Some concerns	No concerns	Major concerns	No concerns	No concerns	Moderate
HB vs TE	Some concerns	Some concerns	No concerns	Major concerns	No concerns	No concerns	Moderate
HY vs TE	Some concerns	Some concerns	No concerns	Major concerns	No concerns	No concerns	Moderate
HB vs UC	Major concerns	Some concerns	No concerns	No concerns	Major concerns	No concerns	Low
**KCCQ**							
CB vs UC	Some concerns	No concerns	No concerns	Some concerns	No concerns	No concerns	High
HB vs UC	Major concerns	No concerns	No concerns	No concerns	Some concerns	No concerns	Moderate
TE vs UC	Major concerns	No concerns	Some concerns	No concerns	Some concerns	No concerns	Moderate
CB vs HB	Some concerns	No concerns	Some concerns	No concerns	Major concerns	No concerns	Moderate
CB vs TE	Major concerns	Some concerns	Some concerns	Major concerns	No concerns	No concerns	Low
HB vs TE	Major concerns	Some concerns	Some concerns	Major concerns	No concerns	No concerns	Low

Tegegne TK, *et al. Open Heart* 2022; 9:e001949. doi: 10.1136/openhrt-2021-001949

**SF-36 mental component**							
CB vs HB	Some concerns	No concerns	No concerns	No concerns	No concerns	No concerns	High
CB vs TE	Major concerns	No concerns	No concerns	No concerns	No concerns	No concerns	Moderate
CB vs UC	Some concerns	No concerns	Some concerns	No concerns	Major concerns	No concerns	Moderate
HB vs TE	Major concerns	No concerns	No concerns	No concerns	No concerns	No concerns	Moderate
HB vs UC	Some concerns	No concerns	Some concerns	No concerns	No concerns	No concerns	High
TE vs UC	Major concerns	No concerns	No concerns	No concerns	No concerns	No concerns	Moderate
**SF-36 physical component**							
CB vs HB	Some concerns	No concerns	No concerns	No concerns	Some concerns	No concerns	High
CB vs TE	Major concerns	No concerns	Some concerns	No concerns	No concerns	No concerns	Moderate
CB vs UC	Some concerns	No concerns	Some concerns	No concerns	Major concerns	No concerns	Moderate
HB vs UC	Some concerns	No concerns	No concerns	No concerns	No concerns	No concerns	High
HB vs TE	Some concerns	No concerns	Some concerns	No concerns	No concerns	No concerns	High
TE vs UC	Major concerns	No concerns	Some concerns	No concerns	No concerns	No concerns	Moderate
**HF-related hospitalization**							
CB vs UC	Some concerns	No concerns	No concerns	No concerns	Some concerns	No concerns	High
HB vs UC	Some concerns	No concerns	No concerns	No concerns	Some concerns	No concerns	High
CB vs HB	Some concerns	No concerns	No concerns	No concerns	Some concerns	No concerns	High
CB vs HY	Some concerns	No concerns	No concerns	Major concerns	No concerns	No concerns	Moderate
CB vs TE	Major concerns	No concerns	No concerns	Some concerns	No concerns	No concerns	Moderate
HB vs TE	Major concerns	No concerns	No concerns	Some concerns	No concerns	No concerns	Moderate
HY vs TE	Major concerns	No concerns	No concerns	Some concerns	Some concerns	No concerns	Moderate
HY vs UC	Major concerns	No concerns	Some concerns	Some concerns	No concerns	No concerns	Moderate

Tegegne TK, *et al. Open Heart* 2022; 9:e001949. doi: 10.1136/openhrt-2021-001949

HB vs HY	Major concerns	No concerns	Some concerns	Major concerns	No concerns	No concerns	Low
TE vs UC	Major concerns	No concerns	Some concerns	No concerns	Major concerns	Some concerns	Low
**HF-related mortality**							
CB vs UC	Major concerns	No concerns	No concerns	No concerns	No concerns	No concerns	Moderate
HB vs UC	Some concerns	No concerns	No concerns	Major concerns	No concerns	No concerns	Moderate
CB vs HB	Some concerns	No concerns	No concerns	Major concerns	No concerns	No concerns	Moderate
HY vs UC	Major concerns	No concerns	No concerns	Major concerns	No concerns	No concerns	Low
HB vs HY	Major concerns	Some concerns	No concerns	Major concerns	No concerns	No concerns	Low
CB vs HY	Major concerns	Some concerns	No concerns	Major concerns	No concerns	No concerns	Low

CB – Centre-based ExCR, HB – Home-based ExCR, HY – Hybrid ExCR, TE – Technology-enabled ExCR and UC – Usual care

GRADE rating [25]:

High —high confidence the true effect is similar to the estimated effect

Moderate —the true effect is probably close to the estimated effect

Low —the true effect may be substantially different from the estimated effect

Very low —the true effect is likely to be substantially different from the estimated effect

Downgrading GRADE domains: No concerns, Some concerns, and Major concerns

1) Within-study bias

In the GRADE assessment table, high risk of bias obtained from the risk of bias assessment was taken as **“Major concerns”**, some concerns as **“Some concerns”**, and low risk of bias as **“No concerns”.**

Tegegne TK, *et al. Open Heart* 2022; 9:e001949. doi: 10.1136/openhrt-2021-001949

2) **Reporting bias**

The “Reporting bias” domain refers to biases that can occur due to publication bias. Judgement of reporting bias was based on asymmetry in funnel plot and Egger’s regression test.

- **No concerns:** No asymmetry in funnel plot and Egger’s test doesn’t indicate funnel plot asymmetry
- **Some concerns:** Asymmetry in funnel plot, but Egger’s test doesn’t indicate funnel plot asymmetry
- **Major concerns:** Asymmetry in funnel plot, and Egger’s test indicates funnel plot asymmetry

3) **Indirectness**

Indirectness is measured based on differences in population/intervention/outcome measurement that could modify treatment effect.

- **No concerns:** No difference in population or intervention or outcome measurement.
- **Some concerns:** If there is difference in one of the three characteristics (study populations, interventions, and outcome measurement)
- **Major concerns:** If there are two or more differences in any combination (study populations, interventions, and outcome measurement)

4) **Imprecision**

Imprecision is assessed by 95% confidence intervals which may include values that could lead to different clinical conclusions. The rules for judging imprecision are based on whether the confidence interval includes the line of no-effect and the clinically important values.

- **No concerns:** Confidence interval lies entirely between the two clinically important values or only include the clinically important value that favours the same intervention as the point estimate
- **Some concerns:** Confidence interval crosses the line of no-effect and extends into clinically important effect
- **Major concerns:** Confidence interval crosses the line of no-effect and extends into clinically important effects in **both** directions

5) **Heterogeneity**

Heterogeneity was judged based on confidence and prediction intervals in relation to the null effect and the clinically important effect on the opposite direction to the point estimate.

- **No concerns:** Confidence and prediction intervals **agree** in relation to clinically important effect
- **Some concerns:** Prediction interval **extends into clinically important or unimportant effects**
- **Major concerns:** Prediction interval extends into clinically important effects in **both** directions

6) **Incoherence**

Incoherence (the disagreement between direct and indirect evidence) was assessed based on three factors: similarity of point estimates, overlap of confidence intervals and statistical test comparing these two estimates. This was determined by the *p* value: if the *p* value is <0.05 then **“Major concerns”**, if between 0.05 and 0.10 then **“Some concerns”**, otherwise **“No concerns”**.

Tegegne TK, *et al. Open Heart* 2022; 9:e001949. doi: 10.1136/openhrt-2021-001949

Supplement 3 League table heatmaps of mean differences and/or odds ratios with 95% credible intervals

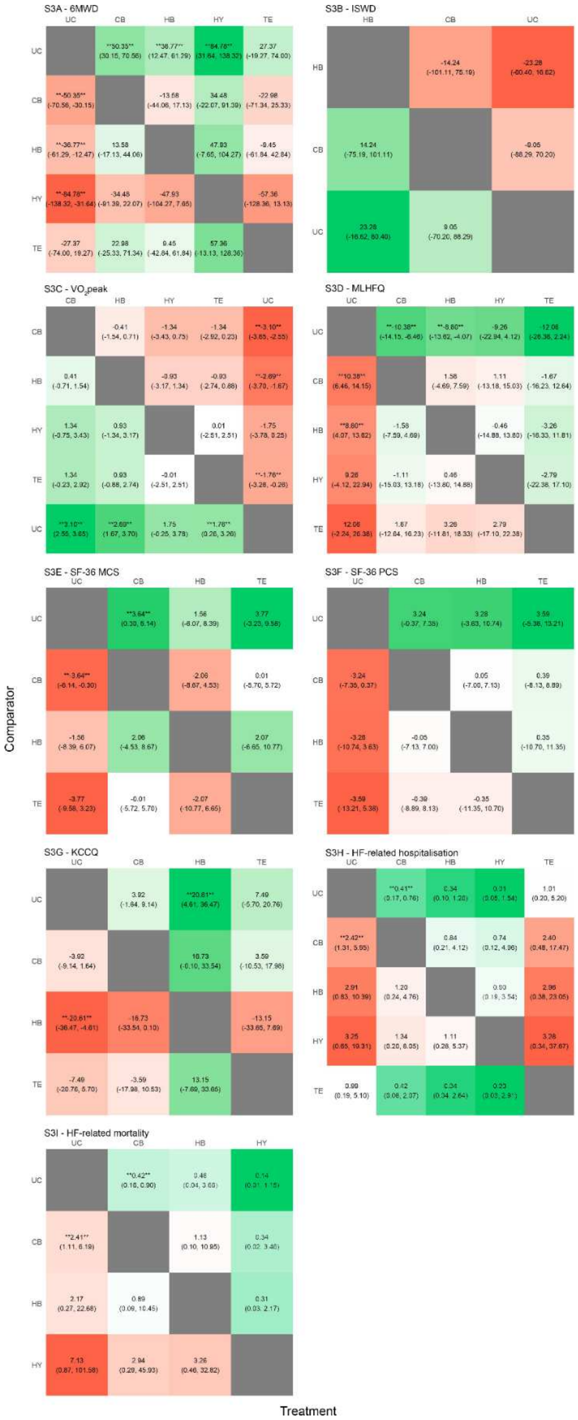

CB – Centre-based ExCR, HB – Home-based ExCR, HY – Hybrid ExCR, TE – Technology-enabled ExCR and UC – Usual care

Green: good effect, White: no effect, Red: bad effect, **: statistically significant (P<0.05)

Supplement 4 Cumulative ranking probability (SUCRA) plots

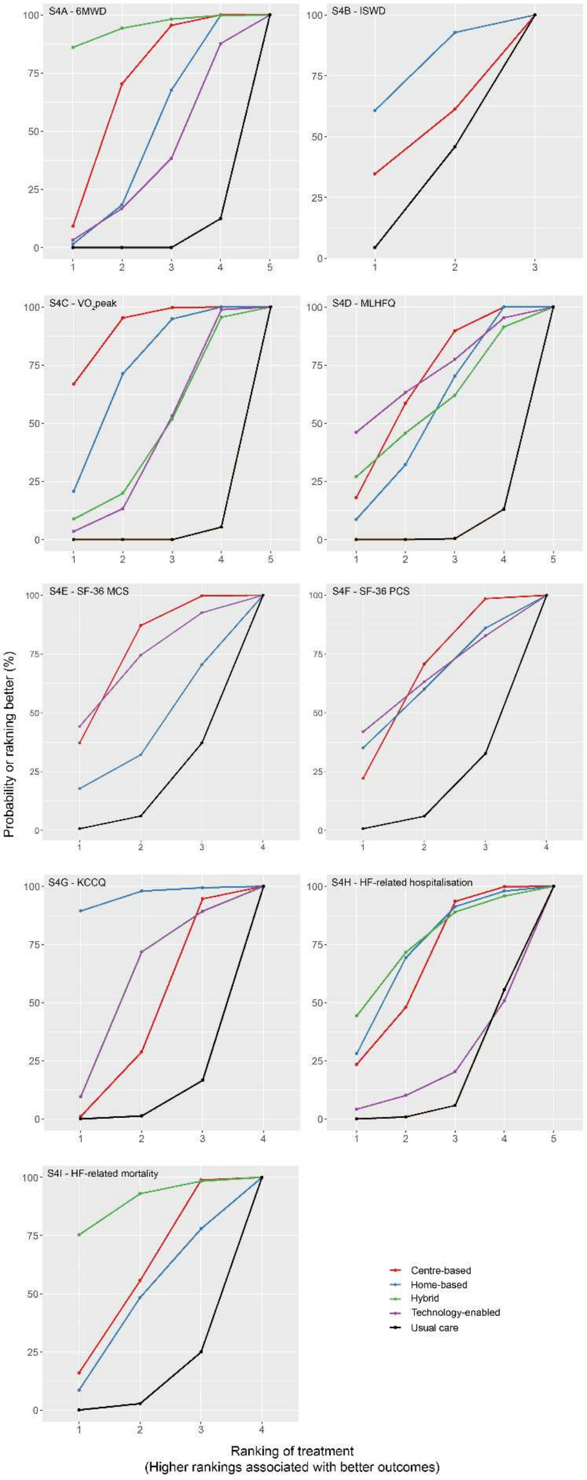

CB – Centre-based ExCR, HB – Home-based ExCR, HY – Hybrid ExCR, TE – Technology-enabled ExCR and UC – Usual care

Tegegne TK, *et al. Open Heart* 2022; 9:e001949. doi: 10.1136/openhrt-2021-001949
